# Newman’s theory of health as expanding consciousness: an evolutionary concept analysis

**DOI:** 10.1186/s12912-024-02262-8

**Published:** 2024-09-03

**Authors:** Hongman Li, Ying Xiong, Zengjie Ye

**Affiliations:** 1 School of Nursing, Guangzhou University of Chinese Medicine, Guangzhou, Guangdong Province China; 2 School of Nursing, Guangzhou Medical University, Guangzhou, Guangdong Province 511436 China

**Keywords:** Health as expanding consciousness, Margaret A. Newman, Rodgers’ evolutionary concept analysis, Theoretical research, Consciousness evolution, Nursing

## Abstract

**Background:**

The health as expanding consciousness (HEC) theory posits that health and disease are interconnected components of a comprehensive process aimed at expanding consciousness.

**Aim:**

The objective of this study is to introduce the concept, research status and applications of HEC and offer a comprehensive understanding of its various key components.

**Data sources:**

Databases including EMBASE, PubMed, ScienceDirect, ProQuest, Wiley, Web of Science, Sinomed, China National Knowledge Infrastructure, Wanfang, and CQVIP, covering the period from 1986 to 2023.

**Method:**

Employing Rodgers’ evolutionary concept analysis approach, this study included and analysed 70 studies.

**Results:**

The characteristics of HEC comprise aspects such as movement, time, space, energy, rhythm, and paradigm of health. The antecedents of HEC encompass disease, chaos, binding, centring, and choice point. Consequences associated with HEC include self-transcendence, unbinding, decentring, expanded consciousness, real freedom, pattern recognition, absolute consciousness, and death.

**Conclusion:**

This study has identified substitute terms, related concepts, attributes, antecedents, consequences, and empirical references associated with HEC. The findings provide valuable information applicable across various domains of nursing, encompassing practice, education, research, and management.

**Supplementary Information:**

The online version contains supplementary material available at 10.1186/s12912-024-02262-8.

## Introduction

Health as expanding consciousness (HEC) represents a theoretical framework developed by Margaret A. Newman [1]. Newman’s scholarly pursuit of nursing was inspired by her experience caring for her mother, who had been diagnosed with amyotrophic lateral sclerosis [2]. During her studies, Newman participated in a seminar on the Rogers’s scientific model of a unitary human being, an experience that led her to recognise the interconnected process of disease and health, from disorder to order [3]. Bohm’s theory further supported Newman’s perspective, emphasising disease as a manifestation of the overall pattern of the individual, elucidating disease as an explication of the underlying implicate pattern of the person [1]. Drawing insights from Prigogine’s theory, Newman comprehended the notion that disruptions in human beings, such as disease or other events, can eventually lead to transformation and a higher level of order, similar to expanded consciousness [4]. Young’s theory of the evolution of consciousness played a crucial role in elaborating on the process of expanding consciousness, fostering the integration of Newman’s previous ideas concerning movement, time, and space. Young’s work contributed to acknowledging the limitations of traditional views on progress and self-development by recognising the point at which “the old rules did not work anymore” [5]. Under the influence of these diverse theoretical foundations, Newman developed and proposed the concept of HEC [6].

Diverging from conventional notions of health, this theory perceives health and disease as essential components of a comprehensive process aimed at expanding consciousness. It posits that health and disease possess dimensional aspects and coexist in a state of relative equilibrium. However, when an individual experiences illness or faces adversity, this equilibrium is disrupted.

The fundamental hypotheses of the HEC theory are as follows: (1) Health encompasses conditions that are described as diseases or pathological conditions within the medical profession. (2) This pathological state is considered as an expression of the individual’s overall pattern. (3) When the personal pattern manifests as a disease, it serves as an initial indication preceding structural and functional changes. Merely eliminating this pathological state does not alter the individual’s pattern. (4) If illness is the sole manifestation of the individual’s pattern, then illness itself becomes a state of health. (5) Health is synonymous with the expansion of consciousness [7, 8].

In the context of nursing, external interventions are employed to help patients to re-evaluate their health or life patterns. The objective is to facilitate the evolution of their consciousness to a higher level and establish a new order that restores equilibrium to their chaotic system. Applying the principles of HEC enables nurses to more effectively assess the underlying needs and issues of patients, create environments conducive to patient recovery, and enhance overall care [5].

Certainly, an inherent limitation of HEC is its abstract nature, making it potentially challenging for individuals to promptly comprehend. When employing the theory as a framework for research, it becomes crucial to establish precise operational and conceptual definitions to prevent any misapplications or misinterpretations.

This study is aimed to introduce the concept, research status, and applications of HEC and offer a comprehensive understanding of its various key components, providing assistance to nurses in applying HEC into nursing practices in clinical settings.

## Methods

### Evolutionary concept analysis method

Rodgers’s evolutionary method [9] was employed in the nursing literature to elucidate the interpretation and significance of Newman’s concept of HEC. The explanatory or descriptive capacities of concepts contribute to the ongoing progression of knowledge. In contrast to conventional methods of concept analysis, the identification or creation of borderline, contrary, invented, and illegitimate cases is now deemed inappropriate. The approach involves identifying surrogate terms, antecedents, consequences, and a model case associated with the concept, with the inclusion of such cases recognised as a significant element in concept analysis methodology [9]. Moreover, this framework offers a viable alternative to a positivist paradigm in conceptual analysis, accommodating diverse findings based on contextual variations. Additionally, it provides an opportunity to identify attributes and associated features in a manner that mitigates biases [10]. The present study employed Rodgers’s evolutionary method, which comprises six phases, for the analysis. (see Table 1) [11].

**Table 1:** Rodgers’ method analysis involves six activities

Stage 1	Identify the concept with associated surrogate terms
Stage 2	Identify and select appropriate setting and sample for data collection
Stage 3	Collect the data (attributes, antecedents, references, surrogate terms, related terms, and consequences)
Stage 4	Analyze the data
Stage 5	Present an exemplar of the concept identified from the literature
Stage 6	Identify the implications for further development of the concept

### Data sources

This analysis aims to explore the application of the concept of HEC, particularly in the context of nursing. A comprehensive search was conducted across various online databases including EMBASE, PubMed, ScienceDirect, ProQuest, Wiley, Web of Science, Sinomed, China National Knowledge Infrastructure, Wanfang, and CQVIP, covering the period from 1986 to 2023. The search terms employed encompassed variations such as “Newman’s theory of health as expanding consciousness”, “health as expanding consciousness”, “Newman’s theory of health expansion”, “nursing praxis within Margaret Newman’s theory of health as expanding consciousness”, “Newman’s theory of HEC”. The Boolean operator “OR” was used to combine the search results. These keywords were searched in the title, abstract, and keyword sections of the studies. A supplementary material shows search strings of databases [see Supplementary Material 1]. After eliminating duplicate studies, 1266 studies remained. The titles of these studies were screened, and book reviews, letters to the editor, irrelevant studies, and studies published in languages other than English or Chinese were excluded. Subsequently, the abstracts of the remaining studies were reviewed. Studies were included in the final analysis if they addressed at least one of the following aspects: surrogate terms and relevant applications, attributes, references, antecedents, consequences, model cases, and implications for the further development of HEC.

**Fig. 1:**
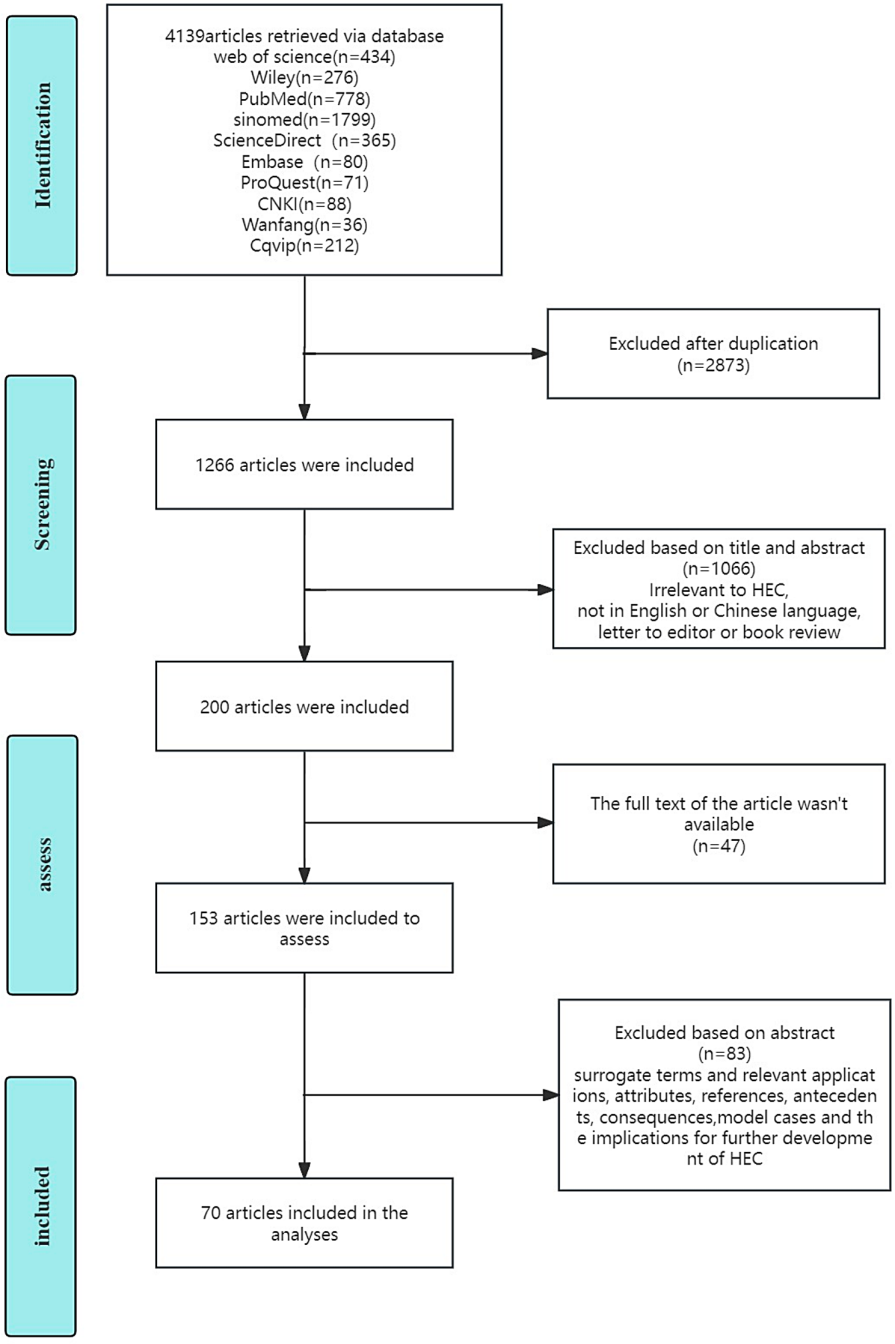
Flowchart illustrating the process of selecting studies for concept analysis

Consequently, 70 studies within the domains of nursing or health sciences were included and analysed (see Fig. 1). Based on Rodgers’s evolutionary method, a literature search was conducted to retrieve publications from the past 3 years, adhering to a minimum requirement of 30 journal articles [12]. Therefore, the selection of 70 articles adhered to Rodgers’s criteria.

## Results

A total of 70 studies were included, and detailed information is presented in Table S1.

### Definition of HEC

HEC involves the process of self-actualisation, discovering deeper meaning in life, and the development of stronger connections with others and the surrounding world [13]. The concept of HEC comprises three subconcepts: health, consciousness, and expansion.

#### Health

According to the Oxford Dictionary (2021), Newman asserts that health refers to the condition of an individual’s body or mind. Health is conceived as a harmonious integration and synthesis of disease and non-disease elements, reflecting the interconnectedness of the entire system. It presents as a holistic pattern of the whole, and the state of wholeness remains constant, though the manifestations of wholeness can manifest in various forms [1, 5]. Furthermore, health is perceived as a transformative process leading to a more comprehensive consciousness [14] and is considered an ongoing process of self-awareness and choice [15]. The theory also posits health as a continuously evolving state encompassing the mind, body, and spirit [4, 16].

#### Consciousness

Consciousness is defined as the informational content of a system and its ability to interact with the environment. Human beings, operating as open energy systems, engage continually with their surroundings [17]. Expanding on this, Newman clarifies that consciousness involves both cognitive and affective awareness, along with the interconnectedness of the entire living system [18]. This interconnectedness encompasses physiochemical maintenance, growth processes, and the immune system [19]. The hypothesis posits that time, space, and movement patterns are correlated with the development of consciousness [20]. Consciousness transcends time and physical space, uniting individuals as part of a greater consciousness that not only brings us together but also extends beyond our collective existence towards a higher consciousness [21]. Throughout life’s journey, various events occur, propelling individuals towards elevated levels of consciousness [1].

#### Expansion

To endure, individuals actively seek novel and varied solutions that reflect a transformation of the self, evolving into a process of inner growth and expansion [22]. The expansion of consciousness is perceived as a process wherein individuals discover deeper meaning in life and establish stronger connections with others and the surrounding world [23]. This process unfolds continuously throughout life, with increasing complexity during successive transitions, particularly through periods of adversity and their subsequent resolution [24]. HEC truly represents a process of “becoming more of oneself, discovering greater meaning in life, and attaining new levels of connectedness with others and the world”. It encompasses personal growth, self-discovery, and a deepening understanding of one’s place within the broader context of existence [25, 26].

### Surrogate terms

Individual concepts might not be exclusively tied to a single specific term but might manifest through several terms. Recognising this variability is crucial for gaining a comprehensive understanding of the concept and its linguistic representation [9]. In the identified studies, surrogate terms related to HEC were HEC and nursing praxis within Margaret Newman’s theory [13, 27]. Specifically, HEC is the abbreviation for “health as expanding consciousness”, while nursing praxis within Margaret Newman’s theory refers to the application of Newman’s theory within the context of nursing practice. The HEC praxis underscores the significance of comprehending the patient as a holistic entity and acknowledging their unique experiences, values, and beliefs [28].

### Related concepts

Related concepts are those that exhibit a degree of association or connection with the primary concept but are not entirely synonymous with it [12].

#### Personal transformation

Throughout history, transformation has been construed as an evolutionary process within consciousness, allowing individuals to perceive the world from a new perspective. This transformative process empowers individuals to attain a clearer and more expansive understanding of the world. Regardless of the level of expanded consciousness, personal transformation involves being aware of one’s consciousness [24]. Indeed, transformation can be considered a preliminary stage of expanding consciousness. Described as a process of fully awakening and unfolding the untapped potential of human consciousness, transformation resembles a continuous journey without a definitive endpoint [28].

#### Allen’s developmental health model

Allen’s developmental health model aims to explore and accumulate knowledge regarding healthy development from the perspective of individuals and families, as well as the role of nursing in facilitating this development [29]. This model particularly underscores competency over deficiency, development over constancy, and process over content. In the context of this model, the family is considered the primary unit of concern, as healthy behaviours are developed within the context of the family [30]. Meanwhile, Newman defines the person as an open energy system that continuously interacts with the environment. Additionally, the person is perceived as an integral part of the life process [31].

#### Young’s theory of the evolution of consciousness

Young’s theory of the evolution of consciousness provides a comprehensive explanation of the process of expanding consciousness [32]. Young’s work highlights the limitations inherent in conventional perspectives on progress and personal development, highlighting a pivotal moment when individuals reach a point where the old rules no longer apply [33]. This phase is referred to as the choice point, marking the need for individuals to transcend the constraints of space and time, entering a realm of higher consciousness that is boundless and timeless [1]. According to Young’s theory, the first stage is binding, characterised by stringent regulations and the sacrifice of individual needs for the collective. The second stage is centring, during which individuals strive for material success and power to establish their identity [34]. The progression continues with the choice stage, prompted by a transformative event revealing that self-determined pursuits are not yielding the desired progress [30]. This juncture demands individuals to explore and understand the operations of this new reality, moving beyond old paradigms towards a reality transcending the limitations of physical space and time [35]. In the decentring stage, individuals shift focus from self-development to broader matters, [36] aiming to create a meaningful life. The subsequent unbinding stage involves the continuous growth of a freedom not bound by time. Finally, in the seventh stage, individuals achieve real freedom, free from any restrictions or constraints [37]. Additionally, Newman integrated her dynamic account of health and life, aligning it with Young’s stage of human evolution (see Table 2) [31].

**Table 2:** The comparison of Young’s theory of human evolution and Newman’s theory of expanding consciousness

stage		Young: human evolution	Newman: expanding consciousness
1		Potential freedom	Potential consciousness
2		Binding	Time
3		Centring	Space
4		Choice	Movement
5		Decentring	Infinite space or boundarylessness
6		Unbinding	Timelessness
7		Real freedom	Absolute consciousness

### Attributes of HEC

Attributes exhibit the most robust relationship with the concept, allowing analysts to gain profound insights [9].

#### Movement, time, and space

Movement is defined as an individual’s capability to foster self-awareness, leading to heighten self-recognition and an expanded state of consciousness [38]. Time is described as a product of movement and a measure of consciousness [20, 39]. In the process of expanding consciousness, freedom from time is experienced. Newman posits that subjective time represents an individual’s perception of time, surpassing objective time [40]. Each individual perceives space differently, with their perception of space rooted in established cognition [41]. The emergence of space represents the initiation of self-awareness and personal growth, resulting in a separation from authority figures. [42]. Movement, time, and space collectively represent the manifestation of expanding consciousness [1].

#### Energy

The individual is perceived as a configuration of energy. While this energy might not directly be observable to the observer, diseases also manifest as patterns of energy [43]. This is evident through observable changes in the patient’s vital signs, such as blood pressure [40].

#### Rhythm

Facial expressions and voice are rhythmic qualities that reflect consciousness [1]. Rhythm plays a significant role in interpersonal relationships, with Newman underscoring the meaningful nature of pauses over the actual spoken words. In nurse-patient interactions, it is crucial to allocate sufficient time for patients to respond, acknowledging the significance of providing space for their thoughts and expressions [40].

#### Paradigm of health

Newman’s perspective on the coexistence of illness and health in varying states and at different times led her to propose the health paradigm. She advocates a shift from merely treating symptoms to a transformative approach, transforming the perception of pain and disease from negative experiences to valuable sources of information [1]. Furthermore, she encourages moving away from viewing individuals as machines that can either be repaired or deemed irreparable, towards recognising them as dynamic energy fields [44]. Newman further emphasises the transition from perceiving diseases as entities to understanding them as processes. This paradigm represents an integrated approach that acknowledges the interconnectedness of various aspects of health and well-being, reflecting a more comprehensive understanding of illness and health [4, 45–47].

### References of HEC

The objective of identifying the references of a concept is to provide clarity regarding the scope of events, situations, or phenomena in which the concept can be appropriately applied [9]. Newman posits that for HEC, disease and health form a cohesive whole, both constituting processes of expanding consciousness. Whether an individual is in an unhealthy state, HEC can make them become more distinctive and actively pursue the meaning of life, thus establishing new connections with the world and reaching higher levels of consciousness. As nurses, the application of HEC involves aiding patients in discovering their potential and advancing to higher levels of consciousness [1, 4, 7, 19, 48–52].

### Antecedents of HEC

The occurrences or phenomena typically observed before an instance of a concept are referred to as its antecedents [9].

#### Disease

Disease and health represent different manifestations of a singular phenomenon, similar to two sides of a coin. They coexist but manifest in different forms based on the timing and conditions. According to Newman, illness serves as an opportunity for individuals to enhance their consciousness, explore their identity, and transform [53]. Illness can be perceived as a “choice point” that motivates individuals to reach higher levels of awareness and facilitates positive change [40].

#### Chaos

Chaotic experiences, as described in this current study, have the potential to continually influence individuals’ lives and their capacity to acknowledge the possibility of change [38]. Disruptions in an individual’s patterns, such as changing circumstances or catastrophic events, frequently serve as catalysts for facilitating a transition from one level of consciousness to a higher level [54].

#### Binding

In the binding phase, individuals tend to focus exclusively on the illness event or other life crisis [32]. Additionally, emphasis is placed on collectivity over individuality, with individuals willing to make sacrifices for the overall well-being of the collective. In this context, individuals have limited autonomy and fewer choices, given the emphasis on collective goals and harmony [55].

#### Centring

In the centring phase, individuals commence a deeper exploration of the underlying processes within the event. They actively seek knowledge to contextualise the crisis events they are undergoing [56]. This phase marks the initial steps in realising that previous coping methods might no longer be appropriate [32].

#### Choice point

The choice point denotes the instance when an individual, confronted with a crisis such as illness, discerns new opportunities and choices that harbour the potential for personal growth [15]. Newman proposed that these periods of disequilibrium are essential evolutionary steps for individuals [57]. This phase is considered a turning point or opportunity to gain insight and transcend limitations, ultimately leading to a state of complete freedom [58, 59].

### Consequences of HEC

The outcomes of a concept are the events that occur as a result of its existence or presence [9].

#### Self-transcendence

Self-transcendence is a crucial element of transitioning towards greater complexity and expanding consciousness. It entails embracing adverse situations that cannot be altered and redirecting one’s focus towards engaging with life optimistically. This emergence of self-transcendence originates from experiences within oneself, with others, and over time, broadening the boundaries of self-consciousness [60]. Characterised by connectedness in both giving and receiving, as well as a sense of hope and personal freedom, self-transcendence holds particular significance in end-of-life issues [4]. As individuals transcend, they have an opportunity for growth by gaining insight and recognising the potential for action [27].

#### Unbinding

Unbinding is the stage during which the individual acknowledges a sense of expanded time and space, along with a feeling of being completely present in the moment [32]. In this phase, the individual experiences an elevated sense of awareness and clarity, facilitating a deeper connection with their surroundings and a more profound engagement [1, 61].

#### Decentring

Decentring is the stage in the process where an individual acknowledges that old ways or patterns of behaviour are no longer beneficial. Having learned new behaviours and experienced self-transcendence, there is a progression towards growth and higher levels of freedom [32]. This phase involves a shift from previous modes of thinking and an openness to new perspectives. The individual starts to adopt a more flexible approach to life and becomes more adaptable to change [61]. Consequently, they are better prepared to navigate challenges and achieve their goals [33].

#### Recognising patterns

Recognising patterns entails comprehending the holistic significance of one’s past, present, and future, providing increased clarity, definition, and direction in the world [33]. Newman proposes that insight is synonymous with pattern recognition [1]. The pattern recognition based on Newman’s theory, termed “caring partnership,” underscores the collaborative and mutually beneficial relationship between the patient and the nurse [62]. In this approach, the nurse acknowledges the patient as an active participant in their care, respecting their autonomy and individuality [63]. The nurse acts as a partner, collaborating with the patient to establish goals, make decisions, and promote the patient’s well-being [64]. This approach extends beyond medical care, encompassing emotional support, empathy, effective communication, and shared decision-making [14]. The caring partnership model aims to foster a therapeutic relationship that enhances the patient’s sense of empowerment, trust, and overall satisfaction with their healthcare experience [65]. Similar to recognising patterns, nursing focuses on individuals’ well-being within their environmental interactions. A fundamental approach involves encouraging clients to discuss the significance of individuals and events in their lives [30]. By actively listening to the client’s narratives, both parties can discern the underlying pattern, representing the manifestation of person-environment interactions [66]. Understanding this underlying pattern facilitates client growth towards heightened levels of consciousness [13, 67].

#### Expanding consciousness

The process of expanding consciousness is characterised by the pursuit of “becoming more of oneself, finding greater meaning in life, and reaching new heights of connectedness with other people and the world” [68, 69]. This process underscores the significance of cultivating a heightened awareness and understanding of one’s true self, fostering a sense of purpose and fulfilment, and establishing meaningful connections with others and the environment. Aligned with the humanistic-existential perspective, it centres on personal growth, self-realisation, and the interconnectedness of individuals within their broader context [13]. Embracing this view of expanding consciousness allows individuals to pursue a more comprehensive and enriching experience of well-being, encompassing physical, emotional, social, and spiritual dimensions.

#### Real freedom

Real freedom denotes a stage in an individual’s experience characterised by unbinding, decentring, higher levels of consciousness, and increased self-awareness. During this stage, individuals can make novel choices and act according to their authentic selves, free from external constraints or social expectations [32]. Real freedom extends beyond the absence of physical or psychological barriers; it encompasses a profound understanding of one’s values, beliefs, and life purpose. This signifies a state of personal growth and self-actualisation, empowering individuals to create a meaningful life in alignment with their true selves. Achieving this stage of real freedom involves various approaches, including personal reflection, spiritual practices, and contemplation of existential themes such as responsibility, meaning, and purpose. Ultimately, it represents a transformational experience that enhances the individual’s quality of life [4, 7, 70].

#### Absolute consciousness

According to Newman, absolute consciousness can be likened to an expansive and limitless deep sea. While it may present a surface of serenity and tranquillity, beneath lies an extensive reservoir of energy and creative potential. This state represents heightened awareness and profound understanding [1]. Newman proposes that life constitutes an ongoing journey, a continual progression towards this infinite capacity. She asserts that humans are in a constant state of evolution, persistently striving for elevated levels of consciousness that perpetually expand [7, 71]. Absolute consciousness is synonymous with love [29].

#### Death

Death does not signify the end of consciousness; instead, it introduces the potential for an elevated state of consciousness, an expansion of awareness, and a deeper understanding of existence [71].

### A model case

The incorporation of a model case is considered a crucial element in concept analysis, with a preference for identifying existing model cases rather than constructing them. These models serve as valuable examples, enhancing the understanding and clarity of a concept.

**Fig. 2:**
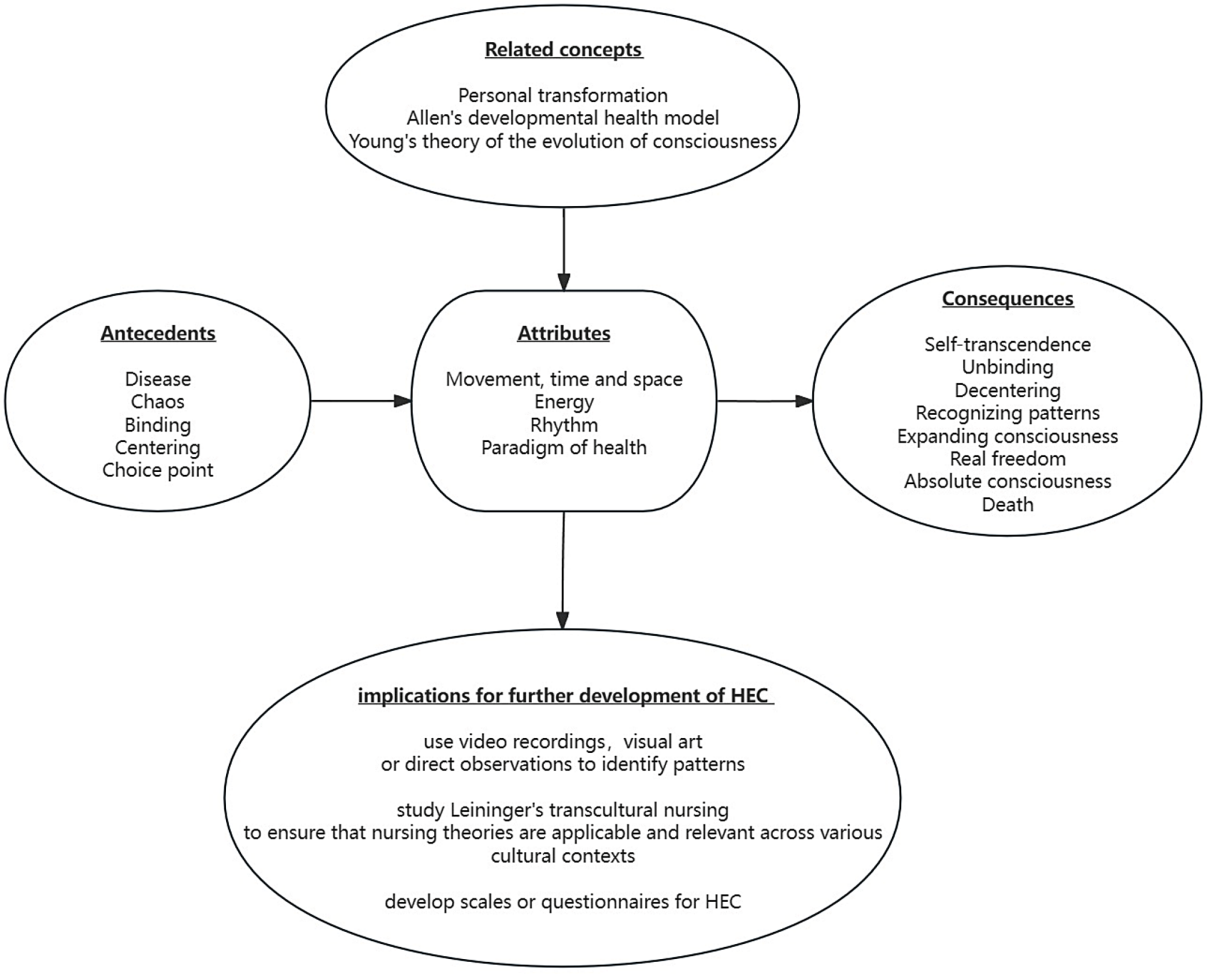
Proposed conceptual model of health as expanding consciousness

In the case of individuals with human immunodeficiency virus during their formative years, an awareness of their distinct sexual orientation was present, yet concealed due to apprehensions about homophobia in mainstream society. Averse to engaging with gay communities or activities, they refrained from disclosing their true orientation, particularly their family, fearing societal non-acceptance. Upon learning about their acquired immunodeficiency syndrome diagnosis, hesitation prevailed in sharing this information, particularly with their mother, amid concerns about causing additional stress. A sense of urgency to prepare for the inevitable ensued. A pivotal transformation occurred when they recognised the significance of their relationships with loved ones, including their parents, friends/partners, and sister, who provided crucial support during the crisis. This support facilitated an expansion of their consciousness, acceptance of their illness, and their taking responsibility for their actions. Peer and family support played a pivotal role in this process. Despite having regrets about past decisions, the assistance of their families, partners, and medical professionals enabled them to ultimately accept their illness. With an expanded consciousness, they regained the confidence to reconnect with their family or chosen family [55, 72]. Figure 2 depicts the conceptual model of HEC developed based on the findings of this study.

### Relationships between each of the concepts

Disease and health represent different manifestations of a singular phenomenon, coexisting but appearing in different forms based on timing and conditions. Chaos manifests as the outward expression of illness while energy signifies its inner manifestation [38]. In the early stages of illness, individuals tend to focus on the events surrounding it and enter a binding phase. As they progress, individuals transition into a centering phase, delving deeper into the underlying processes within the illness event. Illness presents an opportunity for individuals to elevate their consciousness and undergo transformation [73]. It can be seen as a “choice point” that inspires individuals to strive for higher levels of consciousness. Movement, time, and space collectively represent the manifestation of expanding consciousness [1]. This phase is viewed as an experience of unbinding and decentralization, marking a pivotal moment or opportunity to gain insights and self-transcendence. At this stage, nurses use pattern recognition to understand the overall significance of a patient’s past, present, and future. They collaborate with patients to establish goals, make decisions, and thereby help patients elevate to higher levels of consciousness. The process of expanding consciousness is characterized by the pursuit of “becoming more of oneself, finding greater meaning in life, and achieving deeper connections with others and the world” [68, 69], ultimately culminating in a state of true freedom [58, 59]. Life, ultimately, constitutes an ongoing journey—a continual progression towards absolute consciousness [7, 71]. 

### Summary

The HEC theory presents a novel perspective on the relationship between health and disease, asserting that they are a dynamic whole and a process of consciousness expansion—where human life evolves toward higher levels of consciousness [1]. According to this theory, each individual, regardless of apparent illness or despair, is engaged in an overarching process of consciousness expansion. This expansion motivates individuals to actively seek a more meaningful life, thereby enhancing their consciousness [39]. During experiences of disease, disasters, and other challenges, the established old balance could descend into chaos and the old models turn out to be less effective. At the point of making a decision, it is better for individuals to learn new rules or models to establish a new equilibrium and build up better nurse-patient relationship. Newman advocated for nurses to develop deep empathy and establish a positive nurse-patient relationship [15]. Together, nurses and patients confirm old patterns, make choices, discover new patterns and achieve a new equilibrium. Nurses take responsibility for helping patients gain clarity on their physical or mental condition, affirming their current life patterns, guiding them in discovering life’s meaningful aspects through consciousness movements, and facilitating the advancement of their consciousness to higher levels, thereby uncovering their own potential [72]. Throughout this process, both patients and nurses evolve to higher levels of consciousness. This theory draws upon philosophical, psychological, physical, and quantum mechanical foundations and has been found widespread applications across Europe, America, and Asia [39, 47, 56].

## Discussion

This HEC theory offers a fresh perspective on health that challenges conventional views and introduces novel concepts commonly used in nursing theories. The concepts are concise and adaptable to various situations, making them widely relevant and universally applicable [74]. Consequently, the theory has found extensive application in nursing research, development, and clinical practice [6, 74]. The consistent method developed by Newman is used to guide HEC praxis. This method aligns with the unitary, participatory worldview’s ontology and epistemology, ensuring consistency with the understanding of reality and knowledge within this worldview [3].

Newman’s HEC research primarily emphasises qualitative research [14] and conceptual reviews [30, 53, 71]. The research scope has expanded beyond clinical nursing practice to include family and community settings, such as those involving human immunodeficiency virus, [55, 72] rheumatoid arthritis, [24] family caregivers, [54] and nurses, teachers, and students [75, 76]. Community health education now addresses not only clinical diseases but also lifestyle interventions, with a particular emphasis on promoting mental health among patients and enhancing overall quality of life [77].

The necessity for participants to express their patterns verbally poses a valid concern, potentially excluding individuals who are pre-verbal or non-verbal, such as pre-verbal children or individuals who are comatose or unable to speak. In such instances, researchers might need to explore alternative methods to study life patterning [3]. For pre-verbal children, researchers can employ observational methods to examine their behaviours, interactions, and responses to stimuli. This might involve using video recordings or direct observations to identify patterns in their movements, gestures, facial expressions, or other non-verbal cues. Researchers can also engage with parents or caregivers to gather information about the children’s routines, preferences, and experiences. In cases involving individuals who are comatose or unable to speak, studying life patterning becomes more challenging. Researchers might have to rely on indirect measures, such as physiological data, brain imaging, or monitoring devices, to evaluate patterns in bodily functions, neural activity, or responses to stimuli. These methods can offer insights into the individual’s experiences and potential patterns, even in the absence of verbal communication.

Moreover, creative movement and visual art offer alternative avenues to explore life patterning beyond verbal expressions. Researchers can encourage participants to express themselves through dance, body movements, or artistic mediums such as painting, sculpture, or photography. These diverse forms of expression can provide valuable insights into an individual’s experiences, emotions, and patterns of meaning.

Newman’s theory serves as a valuable guide for developing scales and conducting research that combines qualitative and quantitative methods to investigate an individual’s mental state and health patterns. Based on HEC, researchers can develop scales or questionnaires that capture various dimensions of an individual’s mental state and health pattern [6]. These scales can include items related to physical well-being, emotional experiences, social interactions, cognitive processes, and spiritual aspects. Through careful item selection and validation procedures, the scales can provide a quantitative measure of different aspects of the individual’s mental state and health pattern.

Lastly, a diverse and international community of active scholars engaged with Newman’s work spans countries including Australia, Iceland, Korea, Japan, New Zealand, and the United States [3]. This diversity challenges the prevalent practice of importing American nursing theories without considering their cultural appropriateness and sensitivity to diverse contexts. Consequently, it is important for the international team to also study Leininger’s transcultural nursing, which focuses on understanding and providing care for multi-ethnic and culturally diverse groups. This broadened perspective contributes to ensuring the applicability and relevance of nursing theories across various cultural contexts.

## Limitations

This study relies on targeted literature searches using specific terms to guarantee the inclusion of the most relevant literature on HEC. Therefore, it is not feasible to retrieve all articles related to HEC. Moreover, certain articles, particularly the early ones published by Newman, are not accessible in their entirety. Therefore, our understanding of HEC remains incomplete.

## Conclusion

In this study, surrogate terms, related concepts, attributes, antecedents, consequences, and empirical references associated with HEC were identified. The findings of this study hold significant implications for the continued development and refinement of the HEC theory. The analysis of this concept provides valuable information that can be applied across diverse realms of nursing, including practice, education, research, and management.

## Electronic supplementary material

Below is the link to the electronic supplementary material.

Supplementary Material 1

Supplementary Material 2

## Data Availability

No datasets were generated or analysed during the current study.

## Abbreviation

HEC: Health as Expanding Consciousness
