# Supplementary material for: Newman’s theory of health as expanding consciousness: an evolutionary concept analysis
Source: BMC Nurs. 2024 Sep 3;23:616. doi: 10.1186/s12912-024-02262-8 (PMC11373334; doi:10.1186/s12912-024-02262-8)
Supplement: Supplementary file 1 — Supplementary Material 1 [file 12912_2024_2262_MOESM1_ESM.docx]

## Supplementary material 1

## Search strings

### Embase

('newmans theory of health as expanding consciousness':ab,ti OR 'health as expanding consciousness':ab,ti OR 'newmans theory of health expansion':ab,ti OR 'nursing praxis within margaret newmans theory of health as expanding consciousness':ab,ti OR 'newmans theory of hec':ab,ti OR 'theory of health as expanding consciousness':ab,ti OR 'hec theory':ab,ti OR 'newmans theory':ab,ti OR 'expand consciousness':ab,ti) AND [1978-2023]/py

PubMed

((((((((((Newman's theory of health as expanding consciousness[Title/Abstract]) OR (health as expanding consciousness[Title/Abstract])) OR (Newman's theory of health expansion[Title/Abstract])) OR (nursing praxis within Margaret Newman's theory of health as expanding consciousness[Title/Abstract])) OR (Newman's theory of HEC[Title/Abstract])) OR (theory of health as expanding consciousness[Title/Abstract])) OR (HEC theory[Title/Abstract])) OR (Newman's theory[Title/Abstract])) OR (nursing praxis[Title/Abstract])) OR (expand consciousness[Title/Abstract])) AND (("1978"[Date - Publication] : "2023"[Date - Publication]))

ScienceDirect

Title, abstract, keywords:''Newman's theory of health as expanding consciousness'' OR ''health as expanding consciousness'' OR ''Newman's theory of health expansion'' OR ''nursing praxis within Margaret Newman's theory of health as expanding consciousness'' OR ''theory of health as expanding consciousness'' OR ''HEC theory'' OR ''Newman's theory'' OR ''nursing praxis'' OR ''expand consciousness'' AND Year:1978-2023

ProQuest

title(Newman's theory of health as expanding consciousness) OR title(health as expanding consciousness) OR title(Newman's theory of health expansion) OR title(nursing praxis within Margaret Newman's theory of health as expanding consciousness) OR title(Newman's theory of HEC) OR title(theory of health as expanding consciousness) OR title(HEC theory) OR title(Newman's theory) OR title(nursing praxis) OR title(expand consciousness) AND pd(1978-2023)

Wiley

"''Newman's theory of health as expanding consciousness'' OR ''health as expanding consciousness'' OR ''Newman's theory of health expansion'' OR ''nursing praxis within Margaret Newman's theory of health as expanding consciousness'' OR ''Newman's theory of HEC'' OR ''theory of health as expanding consciousness'' OR ''HEC theory'' OR ''Newman's theory'' OR ''nursing praxis'' OR ''expand consciousness''" AND 1978-2023[date]

Web of Science

TI=(Newman's theory of health as expanding consciousness) OR TI=(health as expanding consciousness) OR TI=(Newman's theory of health expansion) OR TI=(nursing praxis within Margaret Newman's theory of health as expanding consciousness) OR TI=(Newman's theory of HEC) OR TI=(theory of health as expanding consciousness) OR TI=(HEC theory) OR TI=(Newman's theory) OR TI=(nursing praxis) OR TI=(expand consciousness) AND DOP=(1978/2023)

Sinomed

(''Newman's theory of health as expanding consciousness'' OR ''health as expanding consciousness'' OR ''Newman's theory of health expansion'' OR ''nursing praxis within Margaret Newman's theory of health as expanding consciousness'' OR ''Newman's theory of HEC'' OR ''theory of health as expanding consciousness'' OR ''HEC theory'' OR ''Newman's theory'' OR ''nursing praxis'' OR ''expand consciousness'') AND 1978-2023[date]

CNKI

(title:"Newman's theory of health as expanding consciousness"(precise)) OR (title: "health as expanding consciousness"(precise)) OR (title:"Newman's theory of health expansion"(precise)) OR (title:"nursingpraxis within Margaret Newman's theory of health as expanding consciousness"(precise)) OR (title:"Newman's theory of HEC"(precise)) OR (title: "theory of health as expanding consciousness"(precise)) OR (title:"HEC theory"(precise)) OR (title:"Newman's theory"(precise)) OR (title: "nursing praxis"(precise)) OR (title: "expand consciousness"(precise)) AND publication date:1978-2023

Wanfang

(title:("Newman's theory of health as expanding consciousness") or title:("health as expanding consciousness") or title:("Newman's theory of health expansion") or title:("nursing praxis within Margaret Newman's theory of health as expanding consciousness") or title:("Newman's theory of HEC") or title:("theory of health as expanding consciousness") or title:("HEC theory") or title:("Newman's theory") or title:("nursing praxis") or title:("expand consciousness")) and publication date:1978-2023

Cqvip

(title:"Newman's theory of health as expanding consciousness"(precise)) OR (title: "health as expanding consciousness"(precise)) OR (title:"Newman's theory of health expansion"(precise)) OR (title:"nursingpraxis within Margaret Newman's theory of health as expanding consciousness"(precise)) OR (title:"Newman's theory of HEC"(precise)) OR (title: "theory of health as expanding consciousness"(precise)) OR (title:"HEC theory"(precise)) OR (title:"Newman's theory"(precise)) OR (title: "nursing praxis"(precise)) OR (title: "expand consciousness"(precise)) AND publication date:1978-2023
